# *Lycium barbarum* Polysaccharide Stabilized Selenium Nanoparticles Exhibit Antibacterial Activity and Ameliorate *Escherichia coli*-Induced Diarrhea in Mice

**DOI:** 10.3390/nano16150901

**Published:** 2026-07-23

**Authors:** Kehan Liu, Wanhe Luo, Md. F. Kulyar, Boyi Zhao, Runjuan Yang, Jiabin Zhang, Ling Zhao, Jianzhong He, Mohammad Mehedi Hasan, Jindong Gao, Mengdi Zhang

**Affiliations:** 1 Engineering Laboratory for Tarim Animal Diseases Diagnosis and Control, College of Animal Science, Tarim University, Alar 843300, China; 3011222602@stumail.taru.edu.cn (K.L.);; 2 Department of Regenerative Medicine, State Research Institute Centre for Innovative Medicine, Santariškių g. 5, LT-08406 Vilnius, Lithuania; muhammad.fakhar@imcentras.lt (M.F.K.);

**Keywords:** *Lycium barbarum* polysaccharides, selenium nanoparticles, *Escherichia coli*, antibiofilm activity, diarrhoea

## Abstract

*Lycium barbarum* polysaccharides are major bioactive constituents of *Lycium barbarum* fruit and are widely recognized for antioxidant, immunomodulatory, and intestinal protective activities. Selenium nanoparticles are considered a safer selenium formulation than conventional inorganic selenium because they generally provide improved bioavailability and reduced toxicity. In this study, *Lycium barbarum* polysaccharide-stabilized selenium nanoparticles were prepared under mild conditions, characterized, and evaluated for antibacterial, antibiofilm, and antidiarrhoeal activities. The optimized nanoparticles exhibited a hydrodynamic diameter of 304.4 ± 32.9 nm, a polydispersity index of 0.369 ± 0.05, and a zeta potential of −20.8 ± 1.5 mV, indicating good colloidal stability. Scanning electron microscopy, Fourier transform infrared spectroscopy, and X-ray photoelectron spectroscopy supported the structural assignment of spherical elemental selenium particles with a polysaccharide-rich surface layer. In vitro assays showed concentration-dependent inhibition of *Escherichia coli*, with minimum inhibitory and minimum bactericidal concentrations of 100 and 200 μg Se/mL, respectively. Biofilm formation was also markedly reduced, and scanning electron microscopy demonstrated severe bacterial surface damage after treatment. In a KM mouse model of *Escherichia coli*-induced diarrhea, oral administration of the nanoparticles significantly lowered diarrhea scores, improved body weight recovery, and alleviated intestinal mucosal injury compared with untreated infected mice. Major organs showed no obvious histopathological abnormalities under the experimental conditions. Overall, these findings identify *Lycium barbarum* polysaccharide-stabilized selenium nanoparticles as a promising selenium-based nanomaterial for controlling bacterial diarrhea and protecting intestinal health. These results provide a practical foundation for future mechanistic, pharmacokinetic, microbiological, and translational studies aimed at broader intestinal applications.

## 1. Introduction

*Lycium barbarum* is a traditional medicinal and edible plant that has long been consumed in East Asia and is increasingly used worldwide in functional food and health-related applications. Among its diverse chemical constituents, *Lycium barbarum* polysaccharides are generally regarded as the principal bioactive fraction. These macromolecules have been associated with antioxidant, immunomodulatory, hepatoprotective, and intestinal protective effects [1], which makes them attractive candidates for designing biologically active delivery systems and biomaterial coatings [2]. In recent years, several groups have further shown that selenium nanoparticles modified or stabilized by *Lycium barbarum*-derived polysaccharides exhibit improved dispersibility, better physicochemical stability, and enhanced biological performance when compared with unstabilized or conventionally formulated selenium systems [3,4,5,6]. This line of work has encouraged the broader exploration of polysaccharide-based selenium nanomaterials for gastrointestinal and antimicrobial applications.

Selenium is an essential trace element that contributes to redox homeostasis, immune regulation, and host defense, mainly through its incorporation into selenoproteins [7]. However, conventional selenium supplements such as selenite and selenate have a comparatively narrow safety window. In practical terms, this limits dose flexibility and raises concerns about toxicity when selenium is used beyond nutritional supplementation. Selenium nanoparticles have therefore attracted substantial interest because they often combine lower toxicity with higher bioavailability and improved biological reactivity relative to inorganic selenium salts [7,8,9]. A growing body of literature indicates that selenium nanoparticles may exert antioxidant, anti-inflammatory, immunomodulatory, and antimicrobial effects, positioning them as promising materials for biomedical, veterinary, and nutraceutical applications [7,10].

Among the different strategies used to improve selenium nanoparticle performance, polysaccharide stabilization is particularly appealing. Polysaccharides can serve as reducing, capping, and protective agents simultaneously, thereby limiting aggregation, improving dispersibility in aqueous systems, and modulating the biological interface of the nanoparticle surface [11]. This is especially relevant in gastrointestinal applications, where particle stability during digestion, mucosal contact, and exposure to changing pH conditions may determine whether a material remains bioactive at the site of action. Previous studies have shown that *Lycium barbarum* polysaccharide-related selenium nanoparticle systems display enhanced colloidal stability, improved anti-fatigue activity, and more favorable behavior during simulated digestion and absorption [12]. Nevertheless, most of the published work has concentrated on physicochemical characterization, antioxidant capacity, or nutritional bioavailability, while direct antibacterial performance and therapeutic relevance in bacterial diarrhea have remained insufficiently investigated.

This knowledge gap is important because bacterial diarrhea caused by pathogenic *Escherichia coli* continues to impose health and economic burdens in both human and animal settings. Recurrent infection, biofilm formation, and the broader challenge of antimicrobial resistance create an urgent need for alternative or adjunctive approaches that go beyond simply suppressing planktonic bacterial growth. A useful candidate would ideally inhibit bacterial proliferation, interfere with biofilm formation, reduce mucosal injury, and support intestinal recovery. Selenium nanoparticles synthesized via multiple routes have been reported to exhibit antibacterial and antibiofilm activity against both Gram-positive and Gram-negative bacteria, including foodborne pathogens and multidrug-resistant *E. coli* [12]. Proposed mechanisms include membrane disruption, oxidative damage, interference with biofilm architecture, and impairment of bacterial growth dynamics [7,12]. These properties make selenium nanomaterials particularly attractive in gastrointestinal infection models, where pathogen control and barrier protection are both relevant.

An additional reason to focus on *Lycium barbarum* polysaccharide-stabilized selenium nanoparticles is the possibility of dual functionality. In earlier work on polysaccharide decorated selenium nanomaterials, anti-inflammatory effects have been documented in intestinal disease models, including attenuation of colitis through inhibition of NF-κB related inflammatory signaling [13]. At the same time, *Lycium barbarum* polysaccharides themselves have been linked to intestinal protective actions, including regulation of mucosal homeostasis and support of gut health [2,3]. If these properties are retained after nanoparticle formation, the resulting material may act through multiple complementary routes: direct antibacterial action from the selenium core and barrier-supportive or immunomodulatory activity from the polysaccharide shell. Such a multifunctional system would be particularly valuable for infectious diarrhoeal conditions in which epithelial injury and inflammation amplify disease severity.

Against this background, the present study was designed to prepare *Lycium barbarum* polysaccharide-stabilized selenium nanoparticles, define their physicochemical characteristics, evaluate their in vitro antibacterial and antibiofilm activity against *Escherichia coli*, and examine their therapeutic efficacy in a KM mouse model of *Escherichia coli*-induced diarrhea. The central hypothesis was that stabilization with *Lycium barbarum* polysaccharides would yield a colloidally stable selenium nanoparticle system with meaningful antibacterial activity and measurable intestinal protective effects in vivo. By testing both in vitro activity and therapeutic response in an animal model, this work seeks to extend earlier physicochemical studies and provide a more application-oriented assessment of LBP SeNPs as a candidate nano selenium preparation for bacterial diarrheal disease.

## 2. Materials and Methods

The study materials were selected to support a reproducible preparation of *Lycium barbarum* polysaccharide-stabilized selenium nanoparticles and to evaluate both physicochemical properties and biological activity. *Lycium barbarum* polysaccharides were purchased from Hefei Bomei Biotechnology Co., Ltd. (Hefei, China) with a stated purity of at least 90% and catalog number B10757. Selenium nanoparticle stock solution was obtained from Zhongke Keyou Co., Ltd. (Beijing, China) at a concentration of 1 mg/mL. The commercial SeNPs were not included in the antibacterial assays because the supplier did not disclose the presence or type of any organic stabilizers or dispersants in the product. Such undisclosed additives could potentially interfere with bacterial growth or biofilm formation, confounding the interpretation of the antibacterial activity. Therefore, all microbiological experiments were performed exclusively with the LBP-SeNPs to ensure that the observed effects could be attributed to the LBP-coated nanoparticles rather than to unknown formulation additives. Crystal violet was purchased from Macklin Biological Co., Ltd. (Shanghai, China), with a reported purity of at least 90%. The *Escherichia coli* isolate used throughout the work was obtained from a clinical infection case at a pig farm in Aksu Prefecture, Xinjiang, and was provided by Wanhe Luo at Tarim University. KM mice were supplied by the Experimental Animal Center of Tarim University in Alar, China. Unless otherwise noted, all solutions were prepared with deionized water.

LBP SeNPs were prepared according to the general approach reported by Liu et al. [4], with optimization guided by dynamic light scattering-derived hydrodynamic diameter, polydispersity index, and zeta potential. In the final optimized formulation, *Lycium barbarum* polysaccharide solution at 4 mg/mL was combined with selenium nanoparticle solution at a volume ratio of 1:4. The mixture was maintained in a constant temperature water bath at 35 °C with continuous stirring for 6 h. These conditions were retained because they yielded the most favorable combination of nanoscale diameter, relatively low dispersity, and sufficiently negative zeta potential, all of which were taken as indicators of improved colloidal stability. After synthesis, the suspension was used for physicochemical characterization and biological experiments. This preparation strategy assumes that the polysaccharide adsorbs to the selenium nanoparticle surface during mixing and incubation, thereby producing a stabilizing organic shell around the selenium core [4].

The morphology of the nanoparticles was examined by scanning electron microscopy using an Apreo S instrument (Thermo Fisher Scientific, Waltham, MA, USA). A drop of nanoparticle suspension was placed onto a silicon wafer, allowed to air-dry, and coated with gold prior to imaging. This analysis was intended to assess overall particle shape, dispersion pattern, and the presence or absence of large aggregates. Hydrodynamic diameter and polydispersity index were measured by dynamic light scattering using a Litesizer (Anton Paar, Graz, Austria) nanoparticle size and zeta potential analyzer, and zeta potential was determined by electrophoretic light scattering on the same instrument. Before measurement, samples were diluted 500-fold with deionized water. Each sample was measured in triplicate, and the results were expressed as mean ± standard deviation. Dynamic light scattering was selected because it provides a convenient estimate of the hydrodynamic particle size in aqueous dispersion, whereas the zeta potential provides indirect information on electrostatic stability. Together, these parameters were used to assess whether polysaccharide stabilization resulted in a practically stable colloidal system.

To investigate the chemical structure and interfacial composition of the particles, Fourier transform infrared spectroscopy and X-ray photoelectron spectroscopy were performed. Fourier (PerkinElmer, Waltham, MA, USA) transform infrared spectra were collected over the range of 4000 to 400 cm^−1^ using the KBr pellet method on a Frontier spectrometer. The purpose of this analysis was to identify major functional groups and detect spectral shifts that might indicate interaction between selenium and the hydroxyl-rich polysaccharide matrix. X-ray photoelectron spectroscopy was then used to determine elemental composition and chemical state. Full survey spectra were collected first, followed by high-resolution spectra for the C 1s, O 1s, and Se 3d regions using a PHI GENESIS 500 X-ray photoelectron spectrometer (Physical Electronics, Chanhassen, MN, USA). Charge correction was referenced to the C 1s peak at 284.8 eV. Deconvolution was performed with appropriate background subtraction and peak fitting constraints based on the reference values for carbon, oxygen, and selenium environments reported in the literature and in commonly used XPS reference resources [14,15,16,17,18,19,20,21,22]. This combined spectroscopic approach was intended to clarify whether the final particles retained elemental selenium and whether the particle surface bore clear evidence of LBP coating.

The in vitro antibacterial activity of LBP SeNPs against *Escherichia coli* was evaluated by determining the minimum inhibitory concentration and minimum bactericidal concentration using a broth microdilution assay. Overnight bacterial culture was adjusted to approximately 1 × 10^6^ CFU/mL in Mueller–Hinton broth. Serial two-fold dilutions of LBP SeNPs were prepared in the same medium across a 96-well microplate. Wells containing bacteria without nanoparticles served as growth controls, and wells containing broth without bacteria served as sterile controls. After incubation at 37 °C for 6 h [23,24,25,26,27], the minimum inhibitory concentration was defined as the lowest LBP-SeNP concentration at which no visible bacterial growth was observed compared with the negative control. To determine the minimum bactericidal concentration, aliquots from wells at or above the minimum inhibitory concentration were subcultured onto Mueller–Hinton agar and incubated at 37 °C for 24 h. The minimum bactericidal concentration was defined as the lowest concentration that resulted in at least a 99.9% reduction in viable colonies relative to the original inoculum. This two-step design enabled the operational distinction between growth inhibition and bacterial killing [12,28].

Antibiofilm activity was assessed by crystal violet staining of attached biomass. *Escherichia coli* suspension at approximately 1 × 10^6^ CFU/mL was incubated in 96-well plates with LBP SeNPs at concentrations ranging from one-quarter to twice the minimum inhibitory concentration. After 24 h at 37 °C, the culture supernatant was removed, and wells were gently washed with phosphate-buffered saline to eliminate non-attached cells. The adherent biofilm was fixed with methanol, stained with 0.1% crystal violet for 20 min, washed thoroughly, and air-dried. Residual bound dye was then quantified at 490 nm after dissolution, and the percentage inhibition of biofilm biomass was calculated relative to the untreated control. This method provided a semi-quantitative measure of the nanoparticles’ effect on the establishment of surface-associated bacterial communities. Because biofilm formation is an important trait associated with persistent infection and antimicrobial tolerance, this assay was considered a necessary complement to planktonic growth inhibition testing [12,28].

To visualize bacterial morphological injury directly, scanning electron microscopy was also applied to untreated and LBP SeNP-treated *Escherichia coli* cells. Bacteria were exposed to LBP SeNPs at twice the minimum inhibitory concentration for 6 h, collected by centrifugation, washed, and fixed overnight in 2.5% glutaraldehyde at 4 °C. After fixation, samples were dehydrated through a graded ethanol series, dried, mounted, and imaged. Untreated bacteria served as the control group. In addition, a bacterial growth curve experiment was conducted to assess whether LBP SeNPs altered the overall proliferation dynamics of *Escherichia coli*. Although the original study focused mainly on descriptive analysis, growth was monitored over time at different nanoparticle concentrations to determine whether treated cultures exhibited delayed logarithmic growth, reduced final density, or both. These complementary methods were used to strengthen the interpretation of the antibacterial phenotype beyond a single endpoint measurement.

For the in vivo study, all animal procedures were reviewed and approved by the Experimental Animal Ethics Committee of Tarim University, Alar, China, under approval number PA20250312053. After 1 week of acclimatization, KM mice were randomly assigned to three groups of six mice each: a normal control group, an *Escherichia coli* model group receiving phosphate-buffered saline after infection, and an *Escherichia coli*-infected treatment group receiving LBP SeNPs. Diarrhea was induced by intragastric administration of *Escherichia coli* suspension at 1 × 10^6^ CFU/mL, 0.2 mL per mouse, once daily for 5 consecutive days. The normal control group received sterile normal saline in place of the bacterial inoculum. Starting from the second day after the final infection challenge, mice in the treatment group were given LBP SeNPs by gavage once daily for 21 days, whereas mice in the model group received an equal volume of phosphate-buffered saline. This design was intended to assess therapeutic rather than purely prophylactic efficacy.

Throughout the animal experiment, body weight, food intake, and general clinical status were monitored daily. Diarrhea severity was evaluated using a semi-quantitative scoring system based on stool consistency and perianal contamination: 0, normal pellets; 1, mildly soft stool; 2, unformed soft stool; 3, watery stool. At the conclusion of the experiment, mice were euthanized, and tissues were collected for gross and microscopic evaluation. The jejunum, ileum, and colon were examined for visible pathological changes, and histopathological analysis was performed after hematoxylin and eosin staining. In addition, the heart, liver, spleen, lung, and kidney were examined histologically to identify any overt treatment-related abnormalities. The inclusion of both intestinal and extraintestinal tissues was important because it allowed the authors to interpret therapeutic efficacy together with preliminary safety observations.

Statistical analysis was conducted using SPSS 27.0.1 (IBM, Armonk, NY, USA). (Statistical Package for the Social Sciences). Quantitative data are presented as mean ± standard deviation. Differences among groups were analyzed using one-way analysis of variance, followed by appropriate post hoc comparisons, and a *p*-value < 0.05 was considered statistically significant. The statistical approach was selected to align with the study’s experimental structure and to enable group-based comparisons of physicochemical measurements, antibacterial endpoints, and animal outcomes.

## 3. Results

**Figure 1:**
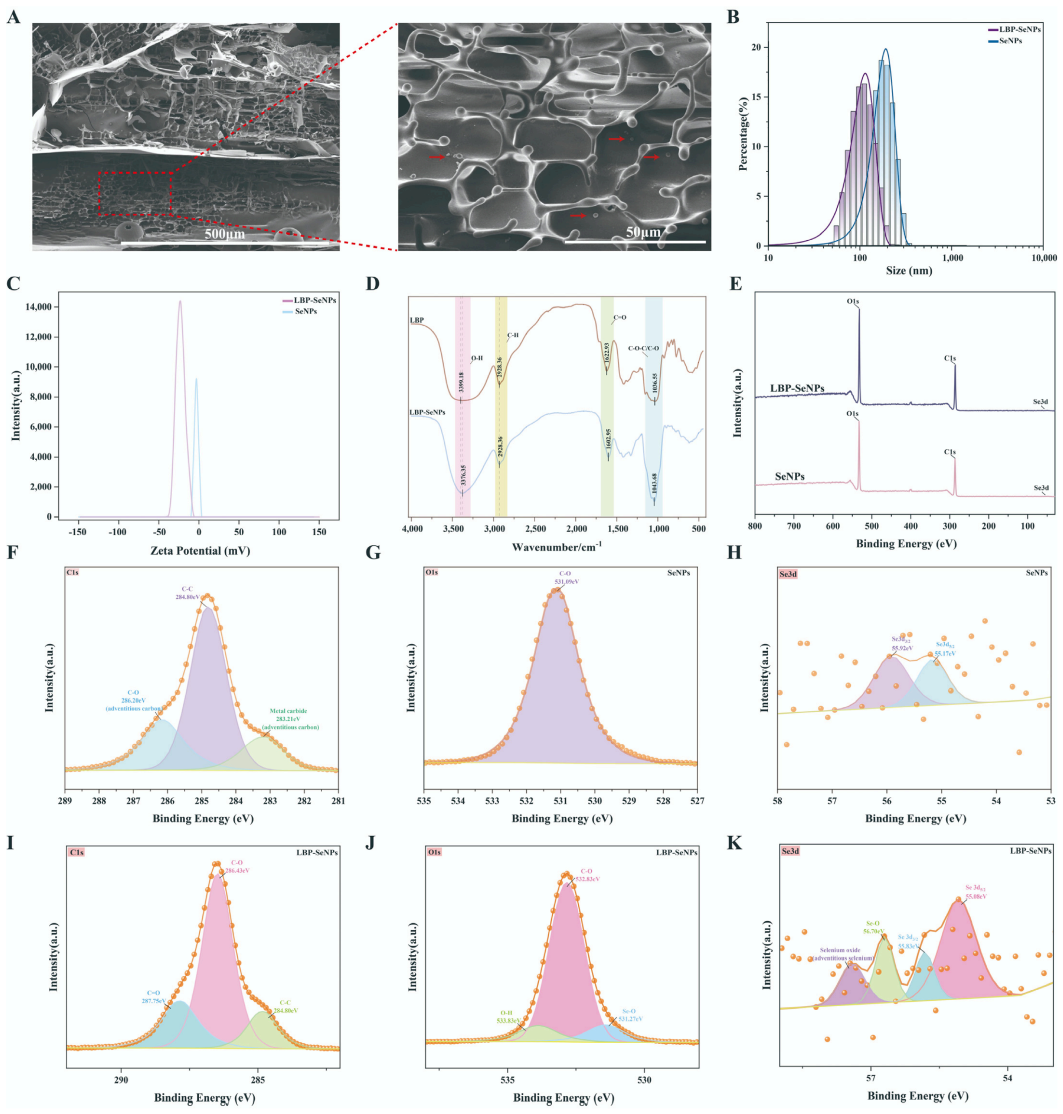
Comprehensive physicochemical characterization of *Lycium barbarum* po/lysaccharide-stabilized selenium nanoparticles. (**A**) Scanning electron microscopy image of LBP SeNPs. (**B**) Hydrodynamic size distribution profiles of LBP and LBP SeNPs. (**C**) Zeta potential of LBP and LBP SeNPs. (**D**) Fourier transform infrared spectra of LBP and LBP SeNPs. (**E**) X ray photoelectron spectroscopy survey spectra of elemental selenium nanoparticles and LBP SeNPs. (**F**–**H**) High-resolution C 1s, O 1s, and Se 3d spectra of elemental selenium nanoparticles. (**I**–**K**) High-resolution C 1s, O 1s, and Se 3d spectra of LBP SeNPs. Note: The label “nano-Se” in the figures has been corrected to “SeNPs” throughout.

Scanning electron microscopy (SEM) images (Figure 1A) show that spherical SeNPs, as indicated by the arrows, are sparsely and uniformly embedded on the polysaccharide surface; individual nanoparticles possess tiny particle sizes without large-scale agglomerates, and their particle sizes are mainly distributed within the range of approximately 200–400 nm, which is basically consistent with the hydrodynamic diameter of 304.4 ± 32.9 nm measured by dynamic light scattering (DLS). Nanoparticles can be clearly distinguished from the silicon wafer substrate in the micrograph: under the secondary electron mode, the nanoparticles exhibit convex spherical morphology with bright signals, while the substrate presents a flat and uniform background contrast. The *Lycium barbarum* polysaccharide (LBP) matrix exerts an outstanding stabilizing and dispersing effect on selenium nanoparticles and effectively inhibits nano-selenium aggregation; the SeNPs are tightly adhered to the polysaccharide skeleton without detached or free large agglomerates, which verifies favorable interfacial compatibility between LBP and SeNPs, confirming that the polysaccharide coating can firmly immobilize elemental selenium nanoparticles and realize homogeneous loading of nanoactive components within the porous biomatrix. Dynamic light scattering further showed that the final formulation had an average hydrodynamic diameter of 304.4 ± 32.9 nm and a polydispersity index of 0.369 ± 0.05, while the measured zeta potential was −20.8 ± 1.5 mV. Taken together, these values indicate a reasonably dispersed colloidal system with moderate uniformity and electrostatic stability. Importantly, the suspension remained macroscopically stable during storage at 4 °C for 30 days, with no visible precipitation or pronounced aggregation. These observations supported the view that *Lycium barbarum* polysaccharides served as an effective stabilizing matrix for selenium nanoparticles and justified the use of the optimized preparation for the subsequent biological assays. The morphology and colloidal properties are illustrated in Figure 1A–C [4,5,6].

Spectroscopic analysis provided complementary evidence for successful surface modification and preservation of elemental selenium. In the X-ray photoelectron spectra of the unmodified selenium nanoparticles, the C 1s region showed components near 284.8, 286.15, and 283.14 eV, whereas the O 1s region was dominated by a single peak near 531.09 eV. Most importantly, the Se 3d spectrum displayed peaks at 55.17 and 55.92 eV, consistent with Se 3d_5_/_2_ and Se 3d_3_/_2_ of elemental selenium. After modification with *Lycium barbarum* polysaccharides, the C 1s spectrum of LBP SeNPs shifted to three principal components at 284.8, 286.28, and 287.75 eV, a pattern consistent with the increased contribution of oxygen-containing species such as CO, COC, and more oxidized carbon environments. Likewise, the O 1s region became more complex, showing components at 531.27, 532.83, and 533.83 eV, which is compatible with chemically heterogeneous oxygen environments introduced by the polysaccharide coating. The Se 3d spectrum of LBP SeNPs contained four peaks at 55.08, 55.83, 56.70, and 57.41 eV. The lower energy doublet remained consistent with elemental selenium, whereas the higher energy components suggested interfacial perturbation of surface selenium atoms rather than persistence of unreacted precursor. Overall, these XPS data supported a core shell type interpretation in which a selenium core remains present but is surrounded by an oxygen-rich organic interface. Figure 1E–K summarize these spectral findings.

The Fourier transform infrared spectra reinforced the XPS observations. Compared with free LBP, the LBP SeNP spectra did not show new major absorption bands, but several existing bands shifted after nanoparticle formation. The broad hydroxyl stretching region moved from 3399.18 cm^−1^ to 3376.36 cm^−1^, and changes were also observed in the carbohydrate fingerprint region and in bands near 1600 to 1650 cm^−1^ and 1400 to 1450 cm^−1^. These shifts indicate that hydroxyl groups and glycosidic oxygen-containing structures in the polysaccharide participated in interfacial interaction with the selenium particle surface. The absence of completely new bands is also noteworthy, because it suggests adsorption and interfacial association rather than formation of an entirely new covalent polymer network.

To further verify the surface state of the raw material, the FTIR spectrum of the unmodified commercial SeNPs was recorded (Figure S1). A weak band at approximately 1043 cm^−11^ was observed; after subtraction of the pure KBr background, this band remained reproducible across repeated measurements. It may be associated with surface oxygen-containing species and/or overlapping C–O-related vibrations from trace organic components [29,30]. Because the XPS data for the commercial SeNPs did not show a resolvable Se(IV) or distinct Se–O component, the exact assignment of this FTIR band remains tentative. Beyond this band, the commercial SeNPs did not show pronounced FTIR features that could be unequivocally assigned to common organic stabilizers, such as aliphatic C–H stretching, amide I/II, or intense glycosidic C–O–C vibrations [31]. However, their FTIR spectrum showed partial similarity to that of LBP-SeNPs [29,30,31,32,33], suggesting that the commercial formulation may contain undisclosed organic stabilizers or dispersants. Therefore, the commercial sample cannot be considered an unequivocally organic-free or chemically defined bare-SeNP preparation. Because the possible presence of undisclosed additives could interfere with bacterial growth or biofilm formation, the commercial SeNP formulation was excluded from the antibacterial and antibiofilm assays. All microbiological experiments were therefore performed exclusively with the LBP-SeNPs to ensure that the observed effects are attributable to the LBP-coated nanoparticles rather than to unknown formulation additives. The Se 3d spectrum of LBP SeNPs contained four peaks at 55.08, 55.83, 56.70, and 57.41 eV. The lower-energy doublet (55.08 and 55.83 eV) remains consistent with elemental selenium (S^00^). The higher-energy components at 56.70 and 57.41 eV are not assigned to selenite (Se(IV)), which would appear at 59–60 eV, but rather to an interfacial perturbation of surface selenium atoms induced by coordination with oxygen-rich functional groups (e.g., hydroxyl and ether oxygens) of the surrounding polysaccharide shell. This interpretation is supported by the concurrent increase in oxygenated carbon species (C 1s at 287.75 eV) and the complexity of the O 1s region (531.27, 532.83, and 533.83 eV), which reflect the polysaccharide coating rather than bulk selenium oxidation. To exclude possible KBr-derived artifacts, we carefully re-evaluated the FTIR data obtained from multiple measurements of the same batch of SeNPs, using the pure KBr background spectrum for subtraction; the ~1043 ^−1^m^−1^ band remained consistent across all spectra and was thus attributed to surface oxygen species rather than to matrix interference. Taken together, the FTIR and XPS results are consistent with a selenium-rich core surrounded by an oxygen-containing organic interfacial layer. However, because EDS elemental mapping and cross-sectional structural characterization were not performed, the core–shell architecture should be regarded as a proposed or plausible structural model rather than definitively demonstrated. These spectral findings are summarized in Figure 1D–K. The observed spectral changes, particularly the shifts in hydroxyl-related FTIR bands and the increased complexity of the C 1s and O 1s XPS regions, are consistent with adsorption of LBP onto the selenium nanoparticle surface via oxygen-rich functional groups, which may contribute to improved stability and altered surface chemistry relevant to biological activity [14,15,16,17,18,19,22].

**Figure 2:**
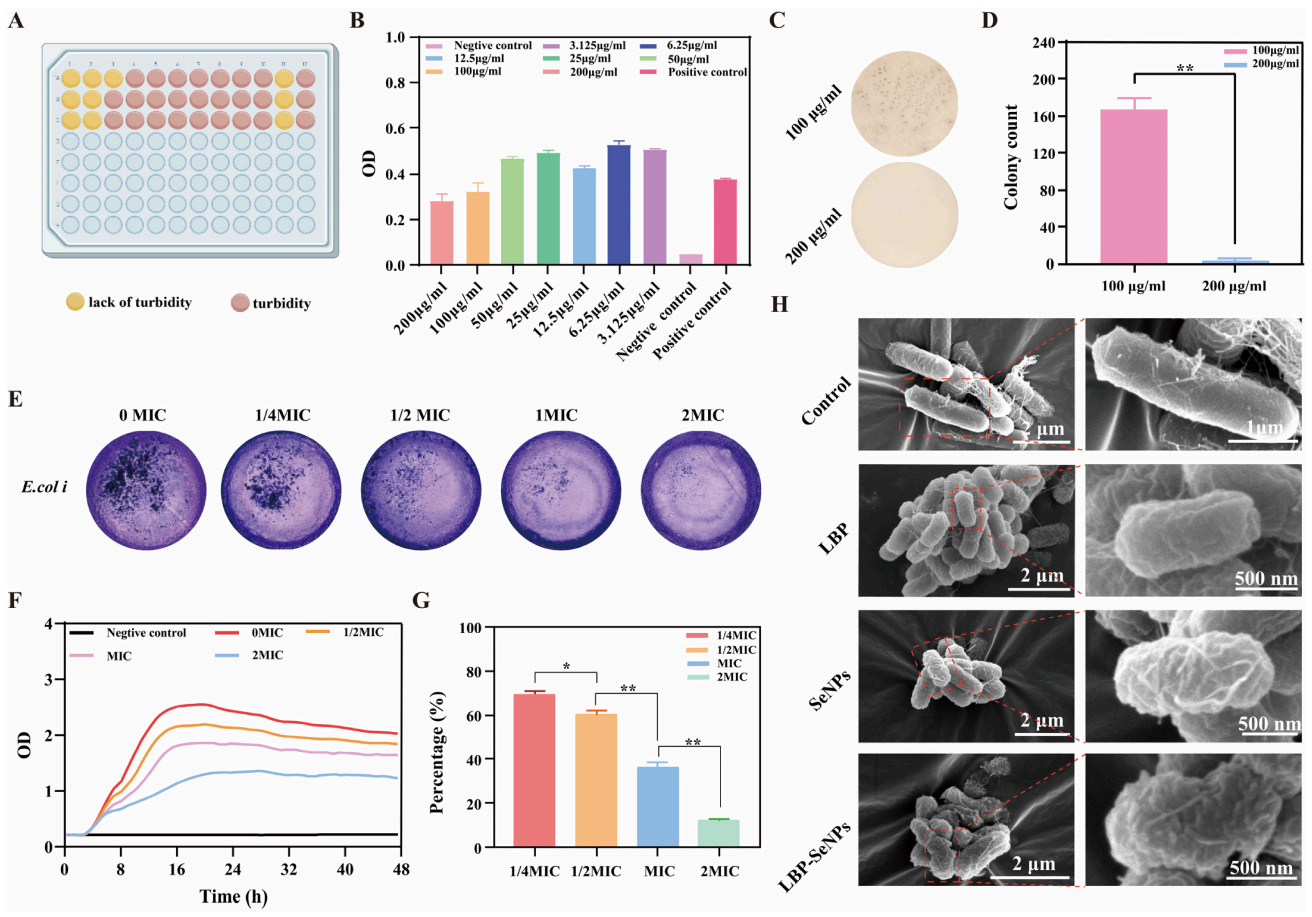
In vitro antibacterial and antibiofilm activities of LBP SeNPs against Escherichia coli. (**A**) Representative broth microdilution assay. (**B**) Minimum inhibitory concentration determination. (**C**) Representative agar subculture plates. (**D**) Minimum bactericidal concentration determination. (**E**) Representative crystal violet-stained biofilms. (**F**) Quantification of biofilm inhibition. (**G**) Bacterial growth curves under different treatment concentrations. (**H**) Scanning electron microscopy images of untreated and LBP SeNP treated bacteria. Data are presented as mean ± SD. * *p* < 0.05, ** *p* < 0.01.

LBP SeNPs showed clear in vitro antibacterial activity against *Escherichia coli*. In broth microdilution testing, bacterial growth inhibition increased with nanoparticle concentration. The minimum inhibitory concentration was 100 μg Se/mL, and the minimum bactericidal concentration was 200 μg Se/mL. Subculture from wells at and above the minimum bactericidal concentration yielded no visible colonies on agar plates, indicating a true bactericidal effect at the higher concentration range. The growth curve analysis supported this interpretation: compared with untreated bacteria, cultures exposed to LBP SeNPs displayed delayed entry into the logarithmic growth phase and a lower final growth density, demonstrating a pronounced concentration-dependent suppression of bacterial proliferation. These observations collectively show that LBP SeNPs affected both short-term visible growth and the pathogen’s broader growth dynamics [12,28]. The antibacterial endpoints are shown in Figure 2A–D,G.

The nanoparticles also markedly inhibited biofilm formation. Crystal violet staining showed that biofilm biomass declined progressively as nanoparticle dose increased. Relative to the untreated control, biofilm formation was reduced by approximately 31.400% at 0.25 × MIC, 39.452% at 0.5 × MIC, 63.504% at 1 × MIC, and 87.594% at 2 × MIC, with statistically significant differences reported at *p* < 0.05. This pattern indicates that the antibiofilm effect was not restricted to concentrations that caused complete planktonic growth arrest but was also evident at subinhibitory levels. Such behavior is important because biofilm-associated infection often persists under conditions in which bacterial growth is only partially restricted. These data suggest that LBP SeNPs interfere not only with bacterial viability but also with the establishment or maintenance of surface-associated bacterial communities [12,28]. The quantitative and representative biofilm data are presented in Figure 2E,F.

Direct microscopic observation of treated bacteria provided morphological evidence consistent with membrane and surface injury. Untreated *Escherichia coli* cells displayed the expected rod-like shape with smooth and intact surfaces. By contrast, bacteria exposed to LBP SeNPs at 2 × MIC showed obvious structural damage, including surface roughening, cell shrinkage, rupture of the cell envelope, and leakage of intracellular material. These findings indicate that one component of the antibacterial mechanism likely involves disruption of the bacterial surface architecture. When interpreted together with the MIC, MBC, growth curve, and biofilm data, the scanning electron microscopy observations support a model in which LBP SeNPs impair the integrity of the bacterial envelope, thereby compromising viability and biofilm development [34]. Representative images are shown in Figure 2H.

**Figure 3:**
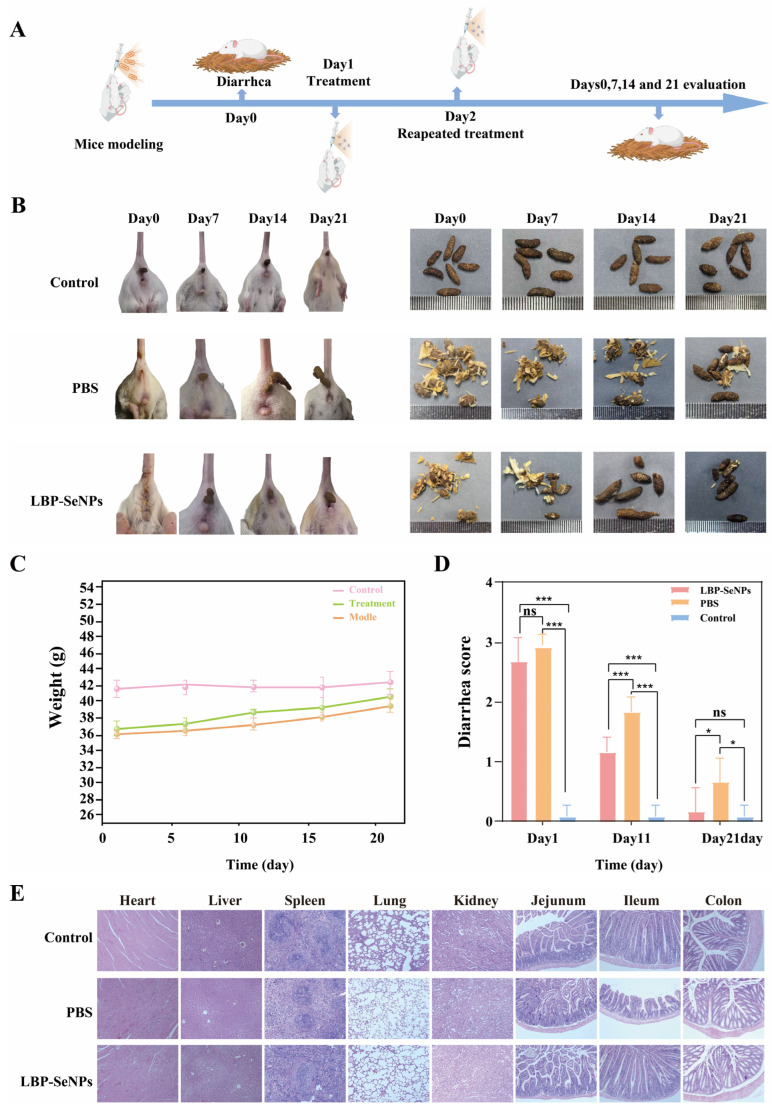
Therapeutic effects of LBP SeNPs in KM mice with Escherichia coli-induced diarrhea. (**A**) Schematic diagram of the animal experiment. (**B**) Representative appearance of mice and faeces during treatment. (**C**) Changes in body weight. (**D**) Diarrhea scores and statistical comparison among groups. (**E**) Hematoxylin and eosin-stained sections of heart, liver, spleen, lung, kidney, jejunum, ileum, and colon from each experimental group. Data are presented as mean ± SD. * *p* < 0.05, *** *p* < 0.001; ns indicates not significant compared with the control group.

The in vivo study showed that LBP SeNP treatment alleviated *Escherichia coli*-induced diarrhea in KM mice. After infection was induced by intragastric challenge for 5 consecutive days, mice receiving phosphate-buffered saline as model treatment developed more severe diarrhoeal symptoms, poorer general condition, and a less favorable body weight trajectory than the healthy control group. In contrast, mice treated orally with LBP SeNPs showed lower diarrhea scores, improved body weight recovery, and better overall appearance than the untreated infected mice. The reduction in diarrhea severity was statistically significant (*p* < 0.05). These findings indicate that the nanoparticle formulation retained therapeutic activity in vivo and that its antibacterial action was sufficiently robust to translate into visible clinical benefit in the experimental diarrhoeal model. The experimental timeline and major clinical observations are summarised in Figure 3A–D.

Histopathological examination further supported the protective effect of treatment. In the healthy control group, the jejunum, ileum, and colon displayed intact mucosal architecture, orderly villi or crypt structures, preserved epithelium, and no obvious inflammatory cell infiltration. In the infected phosphate-buffered saline group, the intestinal tract showed clear pathological injury, including shortened and blunted villi, epithelial disorganization and shedding, crypt disruption, mucosal edema, and inflammatory infiltration, with pronounced lesions especially in the jejunum and ileum. LBP SeNP treatment noticeably improved these histological abnormalities. Treated mice exhibited better preservation of mucosal integrity, improved villus and crypt morphology, and reduced inflammatory infiltration relative to the untreated infected group. In addition, no obvious histopathological abnormalities were observed in the heart, liver, spleen, lung, or kidney under the experimental conditions, providing preliminary support for short-term safety at the administered dose. Representative tissue sections are shown in Figure 3E.

## 4. Discussion

The present study provides a coherent proof of concept that *Lycium barbarum* polysaccharides can be used to generate a selenium nanoparticle formulation with both favorable physicochemical characteristics and meaningful biological activity against *Escherichia coli*-associated intestinal disease. The colloidal data are especially important because the biological usefulness of selenium nanoparticles depends strongly on whether the particles remain dispersed instead of rapidly aggregating. The measured hydrodynamic diameter, moderate polydispersity index, and negative zeta potential suggest that the LBP shell supplied both steric and electrostatic stabilization. This interpretation is consistent with earlier reports showing that *Lycium barbarum*-derived polysaccharides and related conjugates improve the stability and gastrointestinal behavior of selenium nanoparticles [4,6,10,11,35]. Although the hydrodynamic diameter was larger than a dry particle estimate from scanning electron microscopy would suggest, that difference is not unexpected because dynamic light scattering reflects the hydrated particle and its associated surface layer rather than the dry inorganic core alone. On balance, the data support the conclusion that polysaccharide coating successfully converted the selenium dispersion into a more stable and biologically applicable formulation.

The raw commercial SeNPs consisted predominantly of elemental selenium (Se^0^), and no resolvable Se(IV) or distinct Se–O component was detected by XPS within the approximate detection limit of the technique; nevertheless, trace surface oxidation below this limit cannot be ruled out. Given the similarities between their FTIR spectrum and that of LBP-SeNPs, the possible presence of undisclosed additives could not be entirely excluded, which supported the decision to exclude the commercial product from antibacterial assays to avoid potential interference.

The spectroscopic findings strengthen this interpretation. The XPS data support continued predominance of elemental selenium after LBP modification, while the more complex carbon and oxygen signals indicate that the nanoparticle surface became enriched in oxygen-containing organic species. The shifts recorded in the FTIR spectra are consistent with participation of hydroxyl and glycosidic groups in interfacial interactions, although direct evidence of covalent binding was not obtained. When considered together, these results are consistent with a model in which the selenium core is wrapped in a polysaccharide-rich shell, rather than simply mixed with free polysaccharide in solution. That interpretation agrees with published FTIR and XPS analyses of selenium nanoparticles prepared in biological or polysaccharide stabilized systems [14,22]. It is also broadly consistent with established reference assignments for C 1s, O 1s, and Se 3d regions in organic and selenium-containing materials [15,17,18,19,20,21]. One point that warrants careful emphasis is that the higher-binding-energy selenium components in LBP-SeNPs (56.70 and 57.41 eV) are unlikely to represent selenite (Se(IV)), which typically appears at 59–60 eV. Given the absence of a dominant Se(IV) signature and the concurrent enrichment of oxygenated carbon species, a more plausible explanation is an interfacial perturbation of outer-layer selenium atoms induced by coordination with oxygen-rich groups of the surrounding polysaccharide shell. This more cautious interpretation improves the chemical soundness of the structural model.

The antibacterial data indicate that LBP SeNPs act against *Escherichia coli* at multiple levels. First, they inhibited visible planktonic growth and achieved a bactericidal endpoint at twice the minimum inhibitory concentration. Second, they delayed bacterial growth kinetics, showing that exposure reshaped proliferation dynamics even before complete killing occurred. Third, they significantly reduced biofilm formation across subinhibitory and inhibitory concentrations. Fourth, they produced conspicuous morphological disruption of bacterial cells. This multi-level activity is important because biofilm-associated persistence is one reason why intestinal pathogens can be difficult to control. Published studies on selenium nanoparticles have similarly described antibacterial and antibiofilm effects, with mechanisms that may include disruption of membrane integrity, induction of oxidative imbalance, interference with extracellular polymeric matrices, and collapse of normal cellular homeostasis [7,12,28,34].

In the present work, the microscopy findings strongly support cell envelope injury as one important contributor. At the same time, the antibiofilm effect at sub-MIC concentrations suggests that growth inhibition alone does not fully explain the response. The nanoparticle surface, including its polysaccharide shell, may also affect early adhesion events or matrix organization. That possibility should be tested directly in future mechanistic work rather than assumed.

The in vivo data are particularly valuable because they link the in vitro antibacterial phenotype to measurable therapeutic benefit in an intestinal disease model. Oral administration of LBP SeNPs reduced diarrhea severity, improved body weight recovery, and mitigated histopathological injury in the intestine. This pattern is consistent with a dual action model. One part of the effect likely reflects direct suppression of the infecting bacterium, as suggested by the MIC, MBC, growth curve, biofilm, and bacterial scanning electron microscopy results. Another part may reflect support of mucosal recovery through the biological properties of the polysaccharide shell and the gastrointestinal stability of the LBP SeNP system. Previous reports have shown that *Lycium barbarum* polysaccharides can exert beneficial effects on gut homeostasis, and that related polysaccharide-stabilized selenium nanoparticles can maintain useful stability during simulated digestion and absorption [2,5,11]. In addition, other polysaccharide-decorated selenium nanoparticles have shown anti-inflammatory activity in colitis models by modulating NF-κB-associated signaling, which does not allow the individual contributions of selenium, LBP, and their combination to be separated conclusively. That is a strength of the concept, but also a methodological limitation of the current dataset.

Several limitations should be acknowledged in order to place the findings on an appropriate evidential footing. A limitation of the study design is the absence of comparator groups (free LBP, uncoated SeNPs, and physical mixture). Therefore, the individual contributions of selenium, LBP, and their combination cannot be distinguished from the data. The observed antibacterial and therapeutic activities are reported as properties of the LBP-SeNP formulation, without claim of additive or synergistic mechanisms. Bacterial loads in feces and intestinal tissues were not measured. Bacterial translocation to systemic organs was not assessed. Therefore, the therapeutic mechanism underlying the observed improvement in diarrhea symptoms is not fully resolved from these data. Physicochemical characterization was performed in deionized water, not in biorelevant media (MHB, simulated gastric fluid, or simulated intestinal fluid). The colloidal behavior of nanoparticles in these media may differ from that in water; this limits direct extrapolation of the measured parameters to biological conditions. The MIC/MBC endpoints were determined after 6 h of incubation. While this timeframe is supported by published rapid AST methodologies, the CLSI-recommended standard incubation period is 16–24 h. The 6 h protocol used here is an exploratory simplification of the conventional method. Future studies should validate these results using the standard 24 h incubation protocol. In addition, the long-term colloidal stability assessment of LBP-SeNPs in this study has certain limitations. Although macroscopic observation during storage at 4 °C for 30 days showed no visible precipitation or pronounced aggregation, a systematic aging study with periodic DLS monitoring of size distribution, PDI, and zeta potential evolution over time was not performed. Such systematic long-term stability data are critical for the practical application of nanomaterial formulations. Nevertheless, our observations are consistent with previously published reports demonstrating that LBP coating effectively improves the colloidal stability of SeNPs through steric hindrance and electrostatic stabilization mechanisms [4,5,6]. Future studies should systematically evaluate the long-term colloidal stability of LBP-SeNPs under various storage conditions through periodic DLS characterization and zeta potential monitoring. The antibacterial assays relied on a single *Escherichia coli* isolate, so the breadth of activity across other pathogenic or drug-resistant strains remains unknown. The study also did not include comparator groups treated with free LBP alone or uncoated selenium nanoparticles, which means the degree of synergy attributable specifically to polysaccharide stabilization cannot be quantified. Mechanistic biomarkers in vivo, such as inflammatory cytokines, oxidative stress markers, tight junction proteins, intestinal permeability indices, selenium biodistribution, and gut microbiota composition, were not measured. Consequently, the therapeutic mechanism remains plausible but not fully resolved. Finally, safety interpretation should remain cautious. In addition, the SEM characterization in this study has certain limitations. Although the current SEM images clearly demonstrate the spherical morphology, uniform dispersion, and good interfacial binding between LBP and SeNPs, quantitative statistical analysis of particle size distribution using ImageJ software (ImageJ 1.53t (National Institutes of Health, Bethesda, MD, USA; https://imagej.net/ij/, accessed on 20 July 2026)) (e.g., measuring at least 100 particles from multiple fields of view to calculate mean diameter and standard deviation) was not performed; the particle size range was estimated based on visual inspection of representative images. EDS elemental mapping was not obtained under the current experimental conditions, and comparative SEM images of commercial SeNPs were not included. EDS elemental mapping could provide direct evidence for the spatial distribution of selenium and the enrichment of carbon and oxygen in the shell layer, thereby more strongly confirming the core–shell feature of the LBP coating structure. Meanwhile, comparative morphology images of commercial SeNPs and LBP-SeNPs would help visually demonstrate the improvement in particle dispersion and surface morphology conferred by LBP stabilization. Future studies should systematically characterize elemental spatial distribution by EDS mapping and acquire parallel SEM images of uncoated SeNPs to establish direct morphological comparison, which would provide more sufficient microscopic evidence for the confirmation of the LBP shell structure. The absence of overt pathology in major organs is reassuring, yet it does not substitute for a formal toxicological assessment with hematology, serum biochemistry, dose escalation, and longer-term observation. Even with these constraints, the study remains valuable because it moves beyond physicochemical description and demonstrates that LBP SeNPs can connect nanoscale stability with antibacterial performance and therapeutic benefit in a disease-relevant model. The data support continued development, but they should be understood as a strong preclinical starting point rather than a complete translational package.

## 5. Conclusions

This study demonstrates that selenium nanoparticles stabilized with *Lycium barbarum* polysaccharides can be prepared as a stable nanoscale formulation with clear antibacterial and therapeutic potential. The optimized LBP SeNPs showed favorable colloidal properties. The XPS and FTIR data support the proposed core–shell-type model, in which the selenium core remains predominantly in the elemental state and is interfacially associated with an oxygen-rich polysaccharide layer that likely contributes to improved dispersion and bio-interfacial behavior. Biologically, the nanoparticles inhibited *Escherichia coli* growth, suppressed biofilm formation, and induced marked structural damage to bacterial cells in vitro. In the KM mouse model, oral administration alleviated diarrhea, supported body weight recovery, reduced intestinal histopathological injury, and produced no obvious acute tissue abnormalities in major organs under the tested conditions. Taken together, the data support LBP SeNPs as a promising candidate for further development as a selenium-based adjunct for the prevention or management of bacterial diarrhea and related intestinal disorders. Future work should define mechanisms, safety margins, biodistribution, and efficacy across diverse pathogens.

## Data Availability

All data are presented in the manuscript and in Supplementary Information.

## Supplementary Materials

The following supporting information can be downloaded at https://www.mdpi.com/article/10.3390/nano16150901/s1. Figure S1. Fourier transform infrared (FTIR) spectrum of the unmodified commercial selenium nanoparticles (bare SeNPs) used as the raw material. The spectrum does not exhibit strong characteristic absorption bands of common organic stabilizers (e.g., no pronounced C–H at ~2920 cm^−1^, no amide bands at 1640–1550 cm^−1^). The weak band at ~1043 cm^−1^ is attributed to surface oxygen species on the selenium nanoparticles (e.g., O–Se–O vibrations) rather than to KBr-derived interference. Detailed discussion is provided in the main text (Section 3 and Section 4). Figure S2. Overlay of multiple FTIR spectra obtained from different measurements of the same batch of unmodified commercial SeNPs. The spectra show consistent profiles across all measurements, with the weak band at ~1043 cm^−1^ reproducibly present in each scan, confirming that this signal arises from the sample itself (surface oxygen species) rather than from random artifacts or measurement variations. The absence of significant spectral fluctuations supports the reliability of the FTIR data and reinforces the attribution of the ~1043 cm^−1^ band to surface oxygen species rather than to KBr-derived interference. Detailed discussion is provided in the main text (Section 3 and Section 4).
