# Supplementary material for: Lycium barbarum Polysaccharide Stabilized Selenium Nanoparticles Exhibit Antibacterial Activity and Ameliorate Escherichia coli-Induced Diarrhea in Mice
Source: Nanomaterials (Basel). 2026 Jul 23;16(15):901. doi: 10.3390/nano16150901 (PMC13468204; doi:10.3390/nano16150901)
Supplement: Supplementary file 1 [file nanomaterials-16-00901-s001.zip › nanomaterials-4423970-supplementary.pdf]

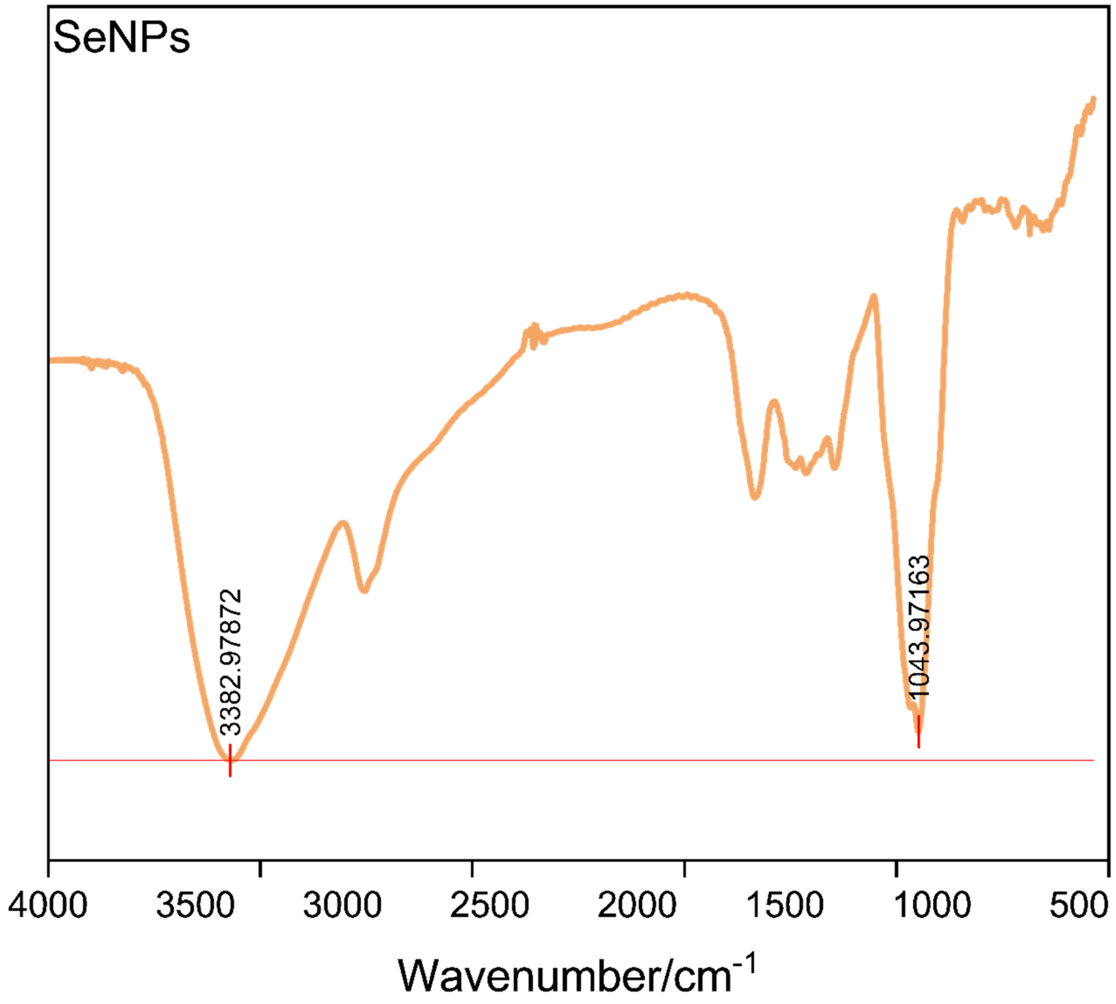

Figure S1. *Fourier transform infrared (FTIR) spectrum of the unmodified commercial selenium nanoparticles (bare SeNPs) used as the raw material. The spectrum does not exhibit strong characteristic absorption bands of common organic stabilizers (e.g., no pronounced C–H at ~2920 cm*$^{-1}$*, no amide bands at 1640–1550 cm*$^{-1}$*). The weak band at ~1043 cm*$^{-1}$ *is attributed to surface oxygen species on the selenium nanoparticles (e.g., O–Se–O vibrations) rather than to KBr-derived interference. Detailed discussion is provided in the main text (Results and Discussion section).*

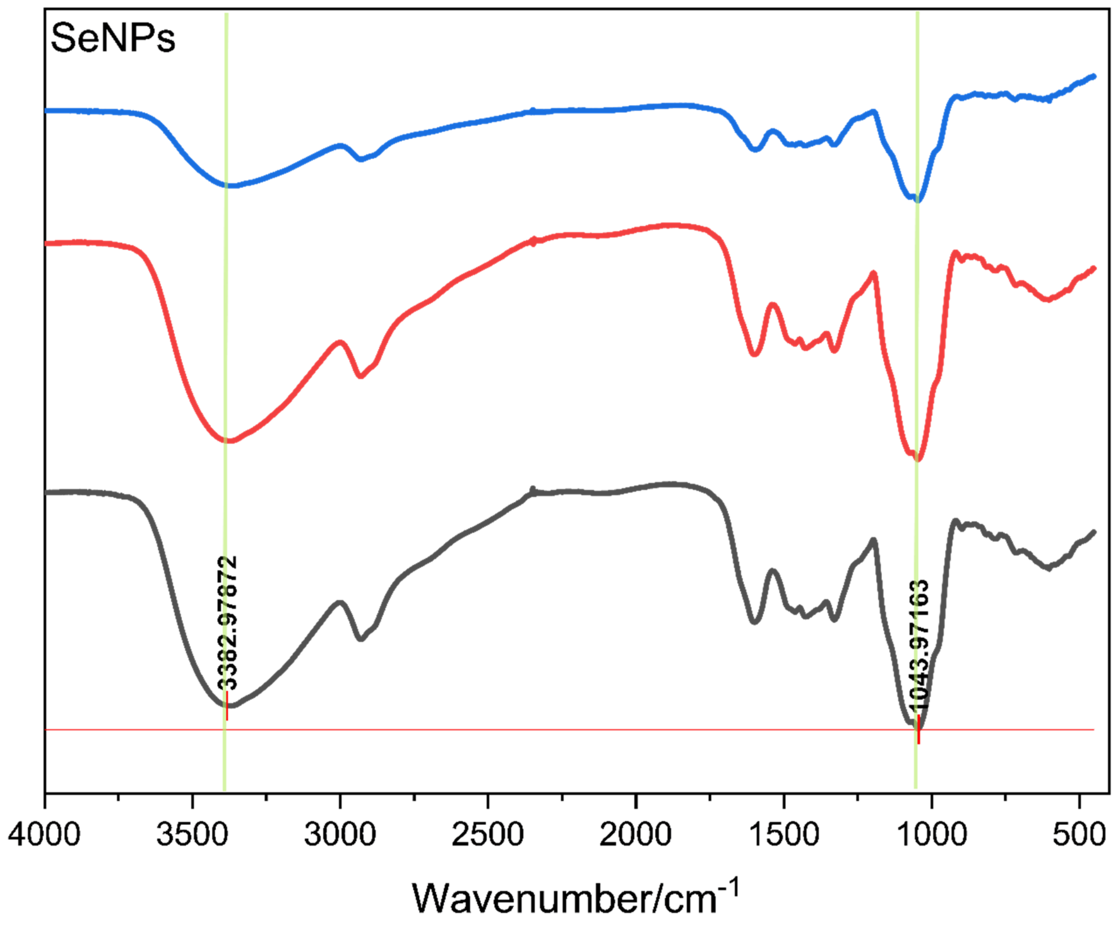

Figure S2. *Overlay of multiple FTIR spectra obtained from different measurements of the same batch of unmodified commercial SeNPs. The spectra show consistent profiles across all measurements, with the weak band at ~1043 cm⁻¹ reproducibly present in each scan, confirming that this signal arises from the sample itself (surface oxygen species) rather than from random artifacts or measurement variations. The absence of significant spectral fluctuations supports the reliability of the FTIR data and reinforces the attribution of the ~1043 cm⁻¹ band to surface oxygen species rather than to KBr-derived interference. Detailed discussion is provided in the main text (Results and Discussion section).*
